# Signal Molecules Mediate the Impact of the Earthworm *Aporrectodea caliginosa* on Growth, Development and Defence of the Plant *Arabidopsis thaliana*

**DOI:** 10.1371/journal.pone.0049504

**Published:** 2012-12-03

**Authors:** Ruben Puga-Freitas, Sébastien Barot, Ludivine Taconnat, Jean-Pierre Renou, Manuel Blouin

**Affiliations:** 1 UMR Biogéochimie et Ecologie des Milieux Continentaux, Université Paris-Est Créteil, Créteil, France; 2 UMR Biogéochimie et Ecologie des Milieux Continentaux, Institut de Recherche pour le Développement, Ecole Normale Supérieure, Paris, France; 3 Unité de Recherche en Génomique Végétale, INRA/CNRS, Evry, France; Wake Forest University, United States of America

## Abstract

Earthworms have generally a positive impact on plant growth, which is often attributed to a trophic mechanism: namely, earthworms increase the release of mineral nutrients from soil litter and organic matter. An alternative hypothesis has been proposed since the discovery of a signal molecule (Indole Acetic Acid) in earthworm faeces. In this study, we used methodologies developed in plant science to gain information on ecological mechanisms involved in plant-earthworm interaction, by looking at plant response to earthworm presence at a molecular level. First, we looked at plant overall response to earthworm faeces in an *in vitro* device where only signal molecules could have an effect on plant growth; we observed that earthworms were inducing positive or negative effects on different plant species. Then, using an *Arabidopsis thaliana* mutant with an impaired auxin transport, we demonstrated the potential of earthworms to stimulate root growth and to revert the dwarf mutant phenotype. Finally, we performed a comparative transcriptomic analysis of *Arabidopsis thaliana* in the presence and absence of earthworms; we found that genes modulated in the presence of earthworms are known to respond to biotic and abiotic stresses, or to the application of exogenous hormones. A comparison of our results with other studies found in databases revealed strong analogies with systemic resistance, induced by signal molecules emitted by Plant Growth Promoting Rhizobacteria and/or elicitors emitted by non-virulent pathogens. Signal molecules such as auxin and ethylene, which are considered as major in plant-microorganisms interactions, can also be of prior importance to explain plant-macroinvertebrates interactions. This could imply revisiting ecological theories which generally stress on the role of trophic relationships.

## Introduction

Plants grow and evolve in close relation with soils and their inhabitants and have done so for several hundred million years. Among soil organisms, earthworms constitute the most abundant animal biomass in terrestrial ecosystems [1]. Reviews of more than 300 assays have revealed that earthworms increase plant growth in 70–80% of cases, with a 56% increase in shoot biomass [2], [3]. The common interpretation of this positive effect is the increased rate of mineral nutrient release from soil litter and organic matter in presence of earthworms. However, this does not provide any satisfying interpretation for cases where earthworms induce a negative effect on plant growth [2], [3], or when they promote an increased plant resistance to parasites [4], [5]. Moreover, earthworms may still increase plant growth even when the soil is supplied with nitrogen amounts higher than needed by the plant [6] and their effects do not necessarily decrease with soil fertility [7].

Alternative interpretations for earthworm effect on plant production have been proposed [3], [8]. Among other hypotheses, signal molecules that mimic plant hormones could be responsible for earthworm impact. Numerous signal molecules can be found in soil. The most widespread are compounds analogous to hormones such as auxins, cytokinins, ethylene, gibberellins, jasmonic and salicylic acids [9]. All these molecules with a basic role in plant growth and development can be synthesized by soil microorganisms. They can have different impact on plants. For example, auxins and cytokinins are said to increase plant growth through the modification of plant morphogenesis, whereas ethylene, salicilic and jasmonic acids induce a resistance to pathogens [10].

These molecules can be produced in soil by microorganisms such as the Plant Growth Promoting Rhizobacteria (PGPR) and may enter into the roots by diffusion or active transporters; although positive effects of PGPR on plants are common, negative effects have also been reported [11]. These bacteria either induce direct changes in plant development thanks to morphogenesis modification, or indirect changes by the biocontrol of plant pathogens or parasites [11], [12]. Transcriptome analyses of *Arabidopsis thaliana* have shown that PGPR may modify the expression of auxin-responsive genes, as well as genes involved in morphogenesis and defence mechanisms [13], [14], [15].

Changes in plant morphogenesis and a higher resistance to pathogens have also been observed in the presence of earthworms [4], [16] or in the presence of the compost they produce [17], [18]. It is acknowledged that humic acids may display hormone-like activity on plant physiology, especially auxin-like effects [19]. This effect was also observed with humic substances derived from earthworm faeces [19], [20]. In addition, indole acetic acid (IAA), which belongs to the auxin family, has already been isolated from humic substances [21], as well as earthworm compost [20]. The origin of these auxin-like compounds is still not well documented, notably due to the difficulty of retrieving and quantifying auxin-like compounds from a natural soil. Whether signal molecules, especially IAA, can be responsible for the positive effect of earthworms on plant growth remains an open issue.

In this work, we used recently developed tools to investigate whether signal molecules could be responsible for earthworm effects on plant growth and development. In this respect, we set up *in vitro* experiments to investigate the potential of earthworm casts to induce significant effects on plant growth via signal molecules. We then focussed on the involvement of molecules related to the auxin signaling pathway by using an *Arabidopsis* mutant with impaired auxin transport. We finally analyzed the transcriptome of *A. thaliana* to identify the main molecular pathways modified in the presence of earthworms and to determine whether these modifications are likely to be caused by signal molecules.

## Results

**Figure 1:**
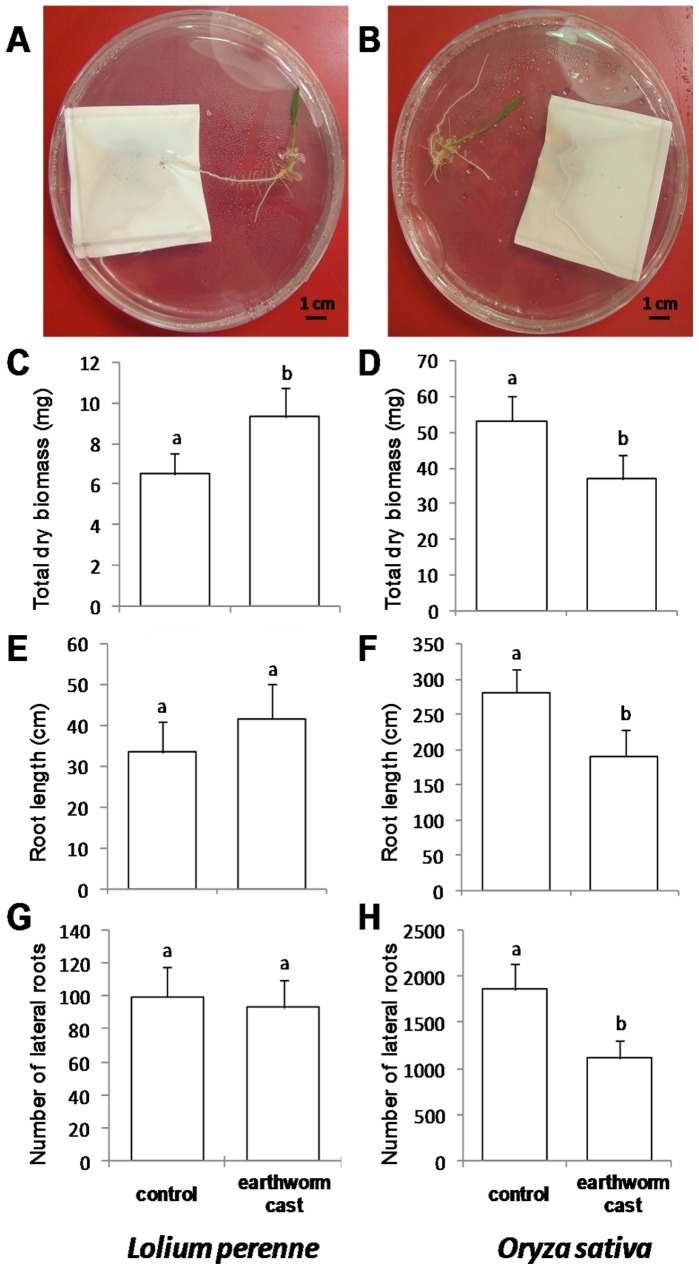
Effect of earthworm casts on plant growth in *in vitro* experiments. Experimental *in vitro* device with *Oryza sativa* in presence of (A) casts of *Aporrectodea caliginosa* or (B) equivalent weight of control soil enclosed into a nylon membrane. Effect of *Aporrectodea caliginosa*’s casts on total biomass production of (C) *Lolium perenne* and (D) *Oryza sativa*. Effect of *Aporrectodea caliginosa*’s casts on root length of (E) *Lolium perenne* and (F) *Oryza sativa*. Effect of *Aporrectodea caliginosa*’s casts on the number of lateral roots of (G) *Lolium perenne* and (H) *Oryza sativa*. Means±s.e., n = 10 per treatment, different letters indicates a significant difference, Tukey HSD, P<0.05.

In the *in vitro* experiments, earthworm casts or non-ingested soil (control) with the same weight were confined in a nylon membrane which prevented the growth of bacteria outside of the nylon bag (Figure 1A and 1B). We observed an increased shoot and total biomass production in *L. perenne* by respectively 50 and 43% in the presence of earthworm casts as compared with control soil (Figure 1C). Root length and the number of lateral roots were not significantly affected (Figure 1E and 1G). Opposite results were obtained with *O. sativa* grown in the same conditions: earthworm casts induced a significant decrease in shoot, root and total biomass by respectively 31, 29 and 30% (Figure 1D). Casts were also responsible for a significant decrease in total root length and the number of lateral roots (Figure 1F and 1H).

**Figure 2:**
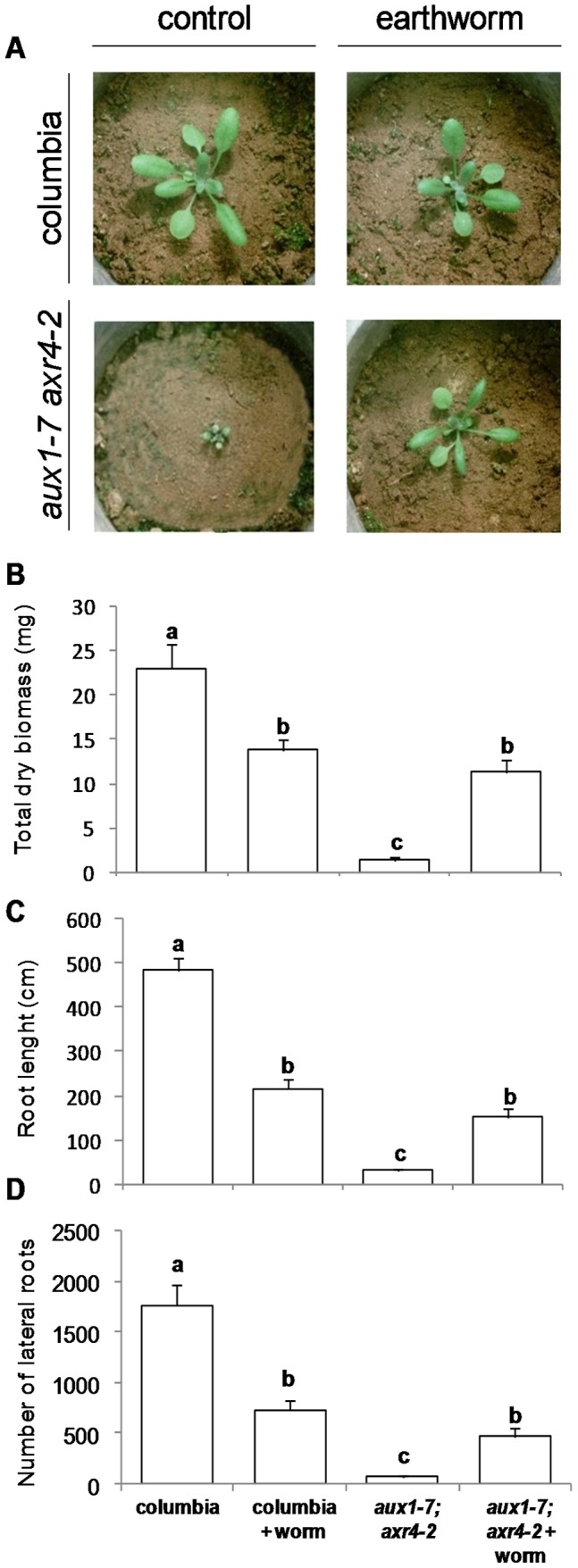
Effects of *Apporectodea caliginosa* on the growth of *Arabidopsis thaliana* cv. Columbia and *aux1-7;axr4-2* mutant. (A) Picture of *Arabidospis thaliana* at three weeks after sowing. Effect of the presence of *Aporrectodea caliginosa* on (B) total biomass production, (C) root length and (D) the number of lateral roots. Means ± s.e., n = 5 per treatment, different letters indicates a significant difference, Tukey HSD, P<0.05.

In a microcosm experiment, we compared the *A. thaliana* response to earthworms casts in wild type and a double mutant with impaired auxin transport (*aux1-7;axr4-2*). The double mutant exhibited a dwarf phenotype in the absence of earthworms: total, aboveground and belowground biomasses were reduced by respectively 94, 94.5 and 86.8% as compared to the wild type (Figure 2A and 2B). The presence of earthworms had a very strong positive effect on the mutant, by increasing total, aboveground and belowground biomasses respectively by 718, 780 and 307% as compared with the mutant without earthworms. Moreover, the mutant exhibited a 4 fold increase in root length and a 6 fold increase in the number of lateral roots (Figure 2C and 2D), parameters which are strongly influenced by auxin and ethylene. All parameters measured on wild type were affected positively in the presence of earthworms (Figure 2A, 2B, 2C and 2D); for example, root length and the number of lateral roots was 2 to 3 times lower. A two-way ANOVA indicated that the interaction between the factors “genotype” and “earthworm” significantly affected all the morphological and growth parameters that we examined (Table 1).

**Table 1:** Impact of the factor “earthworm” and “genotype” on biomass and morphological parameters of *Arabidopsis thaliana*, estimated in a two-ways ANOVA.

	Aboveground dry biomass			Belowground dry biomass			Total dry biomass			Leaf area			Root area		
	Df	F	P-value	Df	F	P-value	Df	F	P-value	Df	F	P-value	Df	F	P-value
**Earthworm**	**1**	**0.08**	**0.78**	**1**	**0.32**	**0.58**	**1**	**0.05**	**0.83**	**1**	**2.68**	**0.12**	**1**	**4.32**	**0.05**
**Genotype**	**1**	**55.3**	**<0.001**	**1**	**16.7**	**<0.001**	**1**	**55.1**	**<0.001**	**1**	**135**	**<0.001**	**1**	**113**	**<0.001**
Earthworm*genotype	**1**	**33.2**	**<0.001**	**1**	**21.7**	**<0.001**	**1**	**34.5**	**<0.001**	**1**	**46.6**	**<0.001**	**1**	**65.7**	**<0.001**
**Residuals**	**16**			**16**			**16**			**16**			**16**		
**n = 20**															

**Figure 3:**
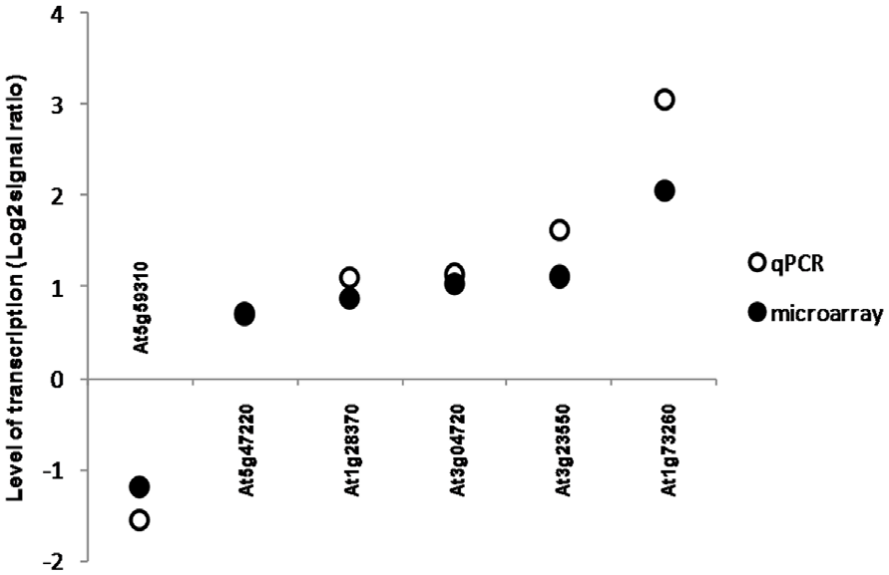
Validation of the results obtained in transcriptomic analysis by real-time polymerase chain reaction (qPCR). Six genes were selected from the 59 differentially expressed genes obtained by transcriptomic analysis (Table 2). Transcript abundance was standardized by reporting it to the constitutive At5g11770 gene. A log base 2 transformation was applied on the average transcript abundance level to obtain data similar to the transformed microarray data.

**Figure 4:**
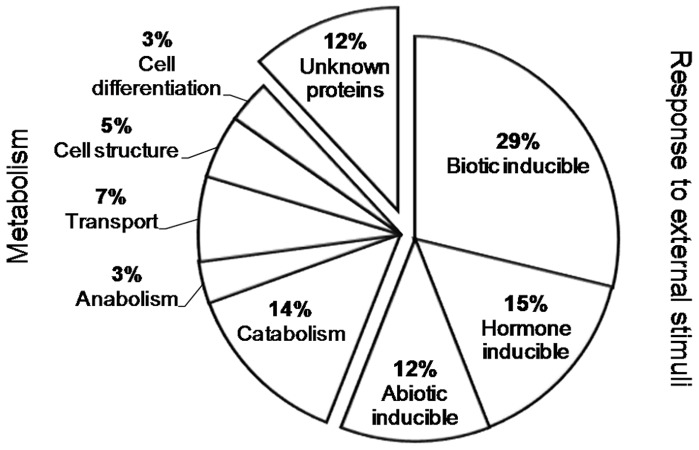
Functions of the 59 *Arabidopsis thaliana* genes differentially expressed in the presence of earthworms. Functional classification was established according to http://www.arabidopsis.org and the related publications.

**Figure 5:**
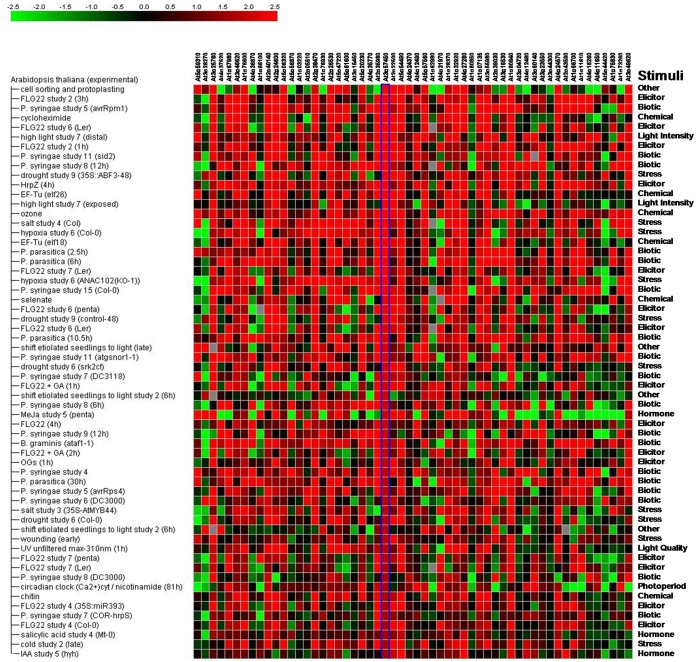
Comparison between genes modulated in the presence of earthworms with other transcriptomic studies. Among our 59 genes, 56 upregulated genes were found in Genvestigator database (in column). We then select a list of 60 studies (in line), among 54 922 referenced in Genevestigator, exhibiting the most similar directional changes with the 56 genes of our study by choosing the “mosaic” with the highest number of red squares (higher transcript abundance in the treatment as compared with control). Green color is corresponding to a lower transcript abundance for the treatment as compared with the control, and red color is corresponding to higher transcript abundance. Color intensity is corresponding to the fold change in gene transcript abundance.

In the third experiment, we studied the effect of earthworms on *Arabidopsis* transcript abundance. After 42 days, plants in the presence of earthworms exhibited no change in leaf area but a 42% increase in above-ground biomass, conversely to our observation in the second experiment with mutants. No significant differences were observed in root system morphology. Results from two repeated experiments conducted under the same conditions with one week of delay were deposited at Gene Expression Omnibus (http://www.ncbi.nlm.nih.gov/geo/, accession no. GSE GSE24393) and at CATdb (http://urgv.evry.inra.fr/CATdb/; Project: AU07-05_ground-worm) according to the “Minimum Information About a Microarray Experiment” standards. We found a significantly modified transcript abundance of 59 genes (Table 2). The transcript abundance of two genes was reduced in the presence of earthworms, whereas transcript abundance of 57 genes was increased. We confirmed by real-time PCR that transcript abundance of a set of six genes was changing in the same direction than with microarrays (Figure 3). The 59 genes are mainly involved in plant interactions with other organisms such as beneficial or pathogenic bacteria (29%), exogenous hormones applications (15%) or abiotic factors (12%) (Figure 4). Some are involved in basal metabolism (32%) and none of them is known to be specific of mineral nutrition. Genes transcripts involved in defense signaling showed increased accumulation such as WRKY33 and WRKY40, involved in the biosynthesis of camalexin (a major phytoalexin which inhibits the growth of pathogens) and induced by salycilic acid and avirulent pathogens [22], [23]. Transcript abundance of Ethylene Response Factors like ERF11; RAP2.9; ERF104 (a nuclear substrate involved in plant defence) and ERF2 known to induce the overexpression of Pathogenesis-related genes such as PR-4 and Plant Defensins PDF1.2 were also differentially accumulated in response to earthworms. Abundance of transcripts of the gene PBP1, coding for a Pinoid Binding Protein was also increased; this gene is known to be up-regulated by auxin [24]. In a same way, transcript abundance of the gene coding for a Lipid Transfer Protein (LTP) which belong to the Pathogenesis-related (PR) proteins was also increased, e.g. genes coding for LTP4 and a another member of the LTP family protein (At4g12490) for which transcript abundance was respectively decreased and increased. Transcript abundance for a gene coding for a putative thionin (At1g66100), which acts synergistically with LTPs for an antifungal activity [25], was increased. Transcripts of a gene coding for a protease inhibitor (At1g73260), the AR781 pheromone receptor [26], a nitrilase responsible for the production of indole-3-acetic acid during bacterial infection [27] (NIT2), a putative chitinase (At2g43590), a lectin like protein whose expression is induced upon treatment with chitin oligomers [28] (At3g16530) and markers for the Hypersensitive Response associated to plant response to pathogen [29] like YLS9 and NHL3 and the transcaffeoyl Coenzyme A 3-O-methyltransferase (At1g67980) involved in the biosynthesis of phenylpropanoid and lignifications which could provide a better plant defence against pathogens by reinforcing cell walls [30] were also over-accumulated. By comparing our list of 59 modulated genes with lists from other studies referenced in Genevestigator, we found many genes in common with studies dealing with biotic factors (30%), elicitors of plant defence (25%) or stress (17%) (Figure 5).

**Table 2:** List of the genes of *Arabidopsis thaliana* differentially expressed in the presence/absence of the earthworm *Aporrectodea caliginosa* in two replicated experiments.

**Locus**	**Function**	**Fold change**	**Pval**	**Locus**	**Function**	**Fold change**	**Pval**
***Down-regulated***				**AT5G54490**	**PBP1 (PINOID-BINDING PROTEIN 1); calcium ion binding**	**0,81**	**2,01E-5**
**AT5G59310**	**LTP4 (LIPID TRANSFER PROTEIN 4); lipid binding**	**−1,18**	**0,0E+**	**AT4G24370**	**unknown protein, phosphorylase family protein**	**0,84**	**3,82E-6**
**AT3G28270**	**unknown protein**	**−0,69**	**3,74E-3**	**AT4G12490**	**(LTP) family protein**	**0,84**	**3,79E-6**
				**AT5G57560**	**TCH4 (TOUCH 4); hydrolase, acting on glycosyl** **bonds**	**0,85**	**2,80E-6**
***Up-regulated***				**AT1G62980**	**ATEXPA18 (ARABIDOPSIS THALIANA** **EXPANSIN A18)**	**0,85**	**2,73E-6**
**AT3G25780**	**AOC3 (ALLENE OXIDE CYCLASE 3)**	**0,63**	**4,65E-2**	**AT4G31970**	**CYP82C2 (cytochrome P450)**	**0,85**	**2,60E-6**
**AT4G37520**	**peroxidase 50 (PER50) (P50) (PRXR2)**	**0,63**	**3,90E-2**	**AT1G28370**	**ATERF11/ERF11 (ERF domain protein 11)**	**0,88**	**4,55E-7**
**AT1G67980**	**CCoAMT (caffeoyl-CoA 3-O-methyltransferase)**	**0,64**	**2,93E-2**	**AT1G32920**	**unknown protein**	**0,88**	**3,67E-7**
**AT4G06746**	**RAP2.9 (related to AP2 9); transcription factor**	**0,65**	**2,14E-2**	**AT4G27280**	**calcium-binding EF hand family protein**	**0,90**	**2,00E-7**
**AT3G46620**	**zinc finger (C3HC4-type RING finger) family protein**	**0,65**	**1,88E-2**	**AT1G80850**	**methyladenine glycosylase family protein,** **WRKY40 (WRKY DNA-binding protein 40);** **transcription factor**	**0,93**	**2,94E-8**
**AT1G76600**	**unknown protein**	**0,66**	**1,42E-2**	**AT1G07135**	**glycine-rich protein**	**0,96**	**6,36E-9**
**AT4G36670**	**mannitol transporter, putative**	**0,67**	**8,59E-3**	**AT3G55980**	**zinc finger (CCCH-type) family protein**	**0,97**	**2,40E-9**
**AT1G66100**	**thionin, putative**	**0,68**	**7,01E-3**	**AT2G39030**	**GCN5-related N-acetyltransferase (GNAT)** **family protein**	**0,99**	**1,15E-9**
**AT2G40140**	**CZF1/ZFAR1**	**0,68**	**4,90E-3**	**AT3G16530**	**legume lectin family protein**	**1,00**	**5,9E-10**
**AT2G24600**	**ankyrin repeat family protein**	**0,69**	**3,95E-3**	**AT1G80840**	**WRKY40**	**1,04**	**6,2E-11**
**AT5G06320**	**NHL3 (NDR1/HIN1-like 3)**	**0,70**	**2,73E-3**	**AT3G04720**	**PR4 (PATHOGENESIS-RELATED 4)**	**1,04**	**4,6E-11**
**AT5G56870**	**beta-galactosidase, putative/lactase, putative**	**0,70**	**2,66E-3**	**AT4G12480**	**pEARLI 1; lipid binding**	**1,09**	**0,0E+0**
**AT1G03220**	**extracellular dermal glycoprotein**	**0,70**	**2,63E-3**	**AT3G60140**	**DIN2 (DARK INDUCIBLE 2**	**1,09**	**0,0E+0**
**AT2G05510**	**glycine-rich protein**	**0,70**	**2,53E-3**	**AT3G23550**	**MATE efflux family protein**	**1,11**	**0,0E+0**
**AT2G38470**	**WRKY33 (WRKY DNA-binding protein 33); transcription factor**	**0,70**	**2,22E-3**	**AT3G44300**	**NIT2 (NITRILASE 2)**	**1,11**	**0,0E+0**
**AT1G76930**	**ATEXT4 (EXTENSIN 4)**	**0,71**	**1,71E-3**	**AT4G24570**	**mitochondrial substrate carrier family protein**	**1,16**	**0,0E+0**
**AT2G26530**	**AR781**	**0,71**	**1,46E-3**	**AT2G43590**	**chitinase, putative**	**1,22**	**0,0E+0**
**AT5G47220**	**ATERF-2/ATERF2/ERF2 (ETHYLENE RESPONSE FACTOR 2)**	**0,71**	**1,39E-3**	**AT1G66700**	**S-adenosyl-L-methionine**	**1,22**	**0,0E+0**
**AT5G61600**	**ethylene-responsive element-binding family protein**	**0,72**	**1,34E-3**	**AT1G11610**	**CYP71A18 (cytochrome P450)**	**1,28**	**0,0E+0**
**AT2G05440**	**glycine-rich protein**	**0,73**	**7,69E-4**	**AT4G16260**	**glycosyl hydrolase family 17 protein**	**1,40**	**0,0E+0**
**AT3G15450**	**unknown protein**	**0,73**	**6,30E-4**	**AT4G11650**	**ATOSM34 (OSMOTIN 34)**	**1,41**	**0,0E+0**
**AT5G20230**	**ATBCB (ARABIDOPSIS BLUE-COPPER-BINDING PROTEIN); copper ion binding**	**0,73**	**5,60E-4**	**AT5G44420**	**PDF1.2 (Low-molecular-weight** **cysteine-rich 77)**	**1,57**	**0,0E+0**
**AT4G35770**	**SEN1 (DARK INDUCIBLE 1)**	**0,74**	**4,68E-4**	**AT3G15356**	**legume lectin family protein**	**1,59**	**0,0E+0**
**AT2G35980**	**YLS9 (YELLOW-LEAF-SPECIFIC GENE 9)**	**0,76**	**2,11E-4**	**AT1G75830**	**LCR67/PDF1.1 (Low-molecular-weight cysteine-rich 67)**	**1,76**	**0,0E+0**
**AT3G57450**	**unknown protein**	**0,78**	**8,01E-5**	**AT1G73260**	**trypsin and protease inhibitor family protein**	**2,05**	**0,0E+0**
**AT2G22500**	**mitochondrial substrate carrier family protein**	**0,80**	**3,17E-5**	**AT3G49620**	**DIN11 (DARK INDUCIBLE 11); oxidoreductase**	**2,38**	**0,0E+0**

The locus column is corresponding to the Arabidopsis Genome Initiative Identification (AGI ID), the function column is corresponding to the identified function reported in publications or putative function from bioinformatic analyses, the fold change column is corresponding to the Log2 of the ratio of transcript abundance in the treatment reported to the transcript abundance in the control, the Pval column is corresponding to the p-value obtained in a paired t-test performed on the log-ratio, adjusted by the Bonferroni method.

## Discussion

In *in vitro* experiments, earthworm casts were enclosed into a nylon membrane, on an agar gel with nutrients *ad libitum*, i.e. at a level which satisfied plant needs. As increased mineralization of organic matter in earthworm casts was negligible compared with nutrient concentrations in the agar gel, observed modifications of plant phenotype could not be due to nutrients diffusing from earthworm casts. No microorganisms could exit the nylon membrane because of the mesh size. Consequently, signaling molecules and other small molecular weight compounds could diffuse from earthworm casts to plant roots, but it is easier to image that signaling molecules or hormones, which act at very low concentrations with huge effects on plant growth and development, are the most likely candidates. In this experimental device, we observed significant positive as well as negative effects, according to plant species. This genotype dependant response is typical of a response to signal molecules, and has already been observed for different rice cultivars exposed to the same earthworm species [31]. Experimental device and results thus converge towards the involvement of signal molecules in the effect of earthworms on plant growth, although other mechanisms can also be important.

**Figure 6:**
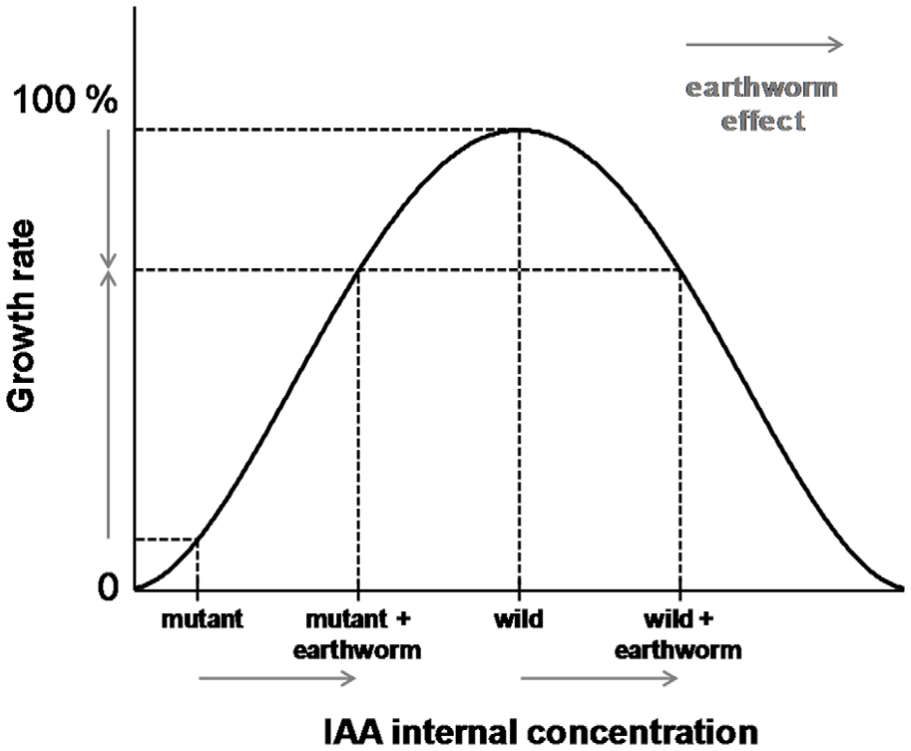
Model explaining contrasting effects of the earthworm *Aporrectodea caliginosa* on *Arabidopsis thaliana*. This model explains positive as well as negative effects on *Arabidopsis thaliana* wild type and mutant for auxin transport (*aux1-7 axr4-2*). Note that the effect of an exogenous auxin supply in the presence of earthworms is dependent on the initial auxin concentration in root cells.

Mutants of *A. thaliana* used in the second experiment had a near-null allele for *AUX1* gene [32], encoding an auxin influx facilitator protein [33], and a null allele for *AXR4* gene [32], encoding for the polar localization of the protein encoded by *AUX1* [34], [35]. Despite a slight auxin production in young roots and an auxin discharge from mature leaves in the phloem [36], impaired auxin transport in mutant is responsible for altered root growth, decreased primary production and dwarf phenotype. The negative effect of earthworms on the wild type and their reversion of the mutant phenotype could be due to either auxin-like compounds, or ethylene, or molecules related to the pathway of one of these hormones. Since this dwarf mutant phenotype is known to be reverted by exogenous auxin application [32] and auxin-like compounds have been isolated from humic substances [21] or earthworm compost [20], earthworms could be responsible for an higher exogenous auxin concentration in soil. This auxin could enter root cells, increase intracellular concentration, and restore altered phenotype of the double mutant. We speculate that in the absence of earthworms, the mutant had too low an auxin concentration in root cells to exhibit the same growth rate as the wild type. In the presence of earthworms, additional exogenous auxin supply increased auxin concentration in root cells, which could be responsible for a higher growth rate (Figure 6). Conversely, auxin elevated above wild-type endogenous levels could lead to an inhibitory effect. This is corroborated by the fact that earthworms stimulate cultivable IAA producing bacteria [37], which could have positive or negative effects on plant growth according to their IAA production level [38], [39].

With regard to the ethylene hypothesis, our transcriptomic analysis revealed the involvement of several ethylene response factors. Ethylene inhibition of root growth in *aux1-7* plants is approximately 30% that of wild type at saturating ethylene concentration [40], and enhanced auxin signaling in root tips after ethylene precursor treatment in the wild type is lost in *aux1* mutant [41]. Auxin and ethylene can have antagonistic effect on lateral root initiation and synergistic effect on root elongation, with reciprocal effects on synthesis and signaling [42]. As a consequence, the fine-tuning of plant growth and development in the presence of earthworms may be due to auxin, ethylene, or the balance between these hormones. Isolation and quantification of auxin or ethylene emissions in soil is particularly difficult due to their low concentration in the large number of biochemicals present in soils. Auxins are adsorbed on organic matter and the number of purification steps can be responsible for significant losses which prevent comparison between treatments. Ethylene would require a specific experimental device to measure gas emissions, and measuring precursor concentration raise the same concerns than auxin. Therefore, we cannot exclude that earthworm effects were due to a molecule acting upstream of auxin and ethylene.

Finally, small molecules recognized as elicitors of plant defence can be responsible for the activation of ethylene signaling pathway. A comparison of our transcriptomic profile with other results published in Genevestigator showed that observed pattern of differences in transcript abundance was typical from a response to flagellin, an activator of plant defence mechanisms against bacteria, or beneficial and non-beneficial bacteria such as many *Pseudomonas syringae* strains (see Results section). Activation of defence mechanisms together with an increased plant biomass are typical of Systemic Acquired Resistance (SAR) triggered by local infection with an incompatible pathogen [43], or eventually of Induced Systemic Resistance (ISR) triggered by many Plant Growth Promoting Rhizobacteria [11]. These mechanisms are known to be under the control of hormones such as ethylene, salicylic acid and jasmonic acid [10].

We observe contrasted effects of the earthworm *Aporrectodea caliginosa* on the growth of *Arabidopsis thaliana* depending on the considered experiment: a negative one on 23 days old plants in the experiment with mutants, but a positive one on 42 days old plants used in transcriptomic analysis. These differences are typical from experiments with earthworms in natural soils: despite numerous precautions to take up the soil at the same place and despite careful homogenization of large soil volumes, there are still residual differences in the physical, chemical or biological properties, especially in bacterial communities, due to initial heterogeneity. Despite this variability, results from our three experiments converge towards the involvement of signal molecules in the effect of earthworms on plants. These molecules could be auxin-like compounds, ethylene, elicitors of plant defence, or a cocktail of these molecules. Novel insights in the signaling networks that regulate synergistic and antagonistic activities of ethylene and auxin [42] and the role of auxin-like compounds or ethylene in SAR [44] would help to choose between different proposed hypotheses.

An ecological perspective to this work could be to explore the parallel between earthworms and PGPR, since both change plant morphogenesis and induce plant resistance to pathogens [11], [13], [14], [15]. This implies to determine whether earthworms (i) are themselves producing signal molecules, (ii) desorb old signal molecules from soil clays and organic matter, (iii) consume and break down bacteria, releasing signal molecules into the soil or (iv) stimulate PGPR or other bacteria producing signal molecules. Recently, it has been shown that the reduction of disease caused by soilborne pathogens by earthworms was associated with the stimulation of *Pseudomonas fluorescens* population, a bacteria recognized as a PGPR [45]. In the same way, protozoa are able to select bacteria producing signal molecules probably by differential grazing [46] and collembola, other soil decomposers, can induce the differential expression of defence and auxin-responsive genes in *A. thaliana* [47]. Despite their long evolutive divergence, earthworms, PGPR, protozoa and collembola seem to modify plant growth through a similar mechanism, i.e. the emission of signal molecules. This strongly encourages research to unravel the potentially common signaling pathway involved in the interaction between plants and soil organisms.

## Materials and Methods

### Soil

Soil was collected at the CEREEP research station (Saint-Pierre-Lès-Nemours, France) in a natural meadow, with the permission of Beatriz Decencière, project coordinator of the CEREEP - Ecotron IDF/UMS CNRS/ENS 3194. It was dried at 25°C for a week and sieved at 2 mm mesh size. This soil has the following properties: total organic carbon content, 14.7 g kg^−1^; total nitrogen content, 1.19 g kg^−1^; pH, 5.22; CEC, 4.08 cmol kg^−1^; texture: 6.9% clay, 19.0% silt, 74.1% sand.

### Earthworms

Adults of *Aporrectodea caliginosa* Savigny (Annelida, Oligochaeta) were retrieved with permission at the CEREEP - Ecotron IDF. It is an endogeic earthworm which makes horizontal or randomly oriented burrows, considered to be temporary structures because they are rarely reused. For the *in vitro* experiment, two breeding boxes were prepared using the same weight of soil maintained at 80% of the field capacity. Earthworms were added in one of these boxes, the other one being used as control soil. After 14 days of incubation, earthworm feces, also called casts, and control soil were retrieved from their respective breeding boxes. In the experiments dedicated to transcriptome analysis or to the study of the double mutant, living earthworms were added to the devoted microcosms at a density close to the one observed in the field in France [1].

### *In vitro* Experiment

Plants were grown in sterile Petri dishes (14 cm diameter, 20.6 mm height, Fisher Scientific, France) in the presence of a non-sterile earthworm casts or same weight of control soil enclosed in a nylon membrane (Figure 1A). Culture medium was made of 7 g l^−1^ gelrite (Duchefa Biochemie, U.S.A.). Nutrients were supplied at 4.3 g l^−1^ of basal salt mixture (Duchefa Biochemie, U.S.A.), according to the well known Murashige and Skoog plant culture media [48]. Macronutrients were supplied as follow: CaCl_2_∶ 332 mg l^−1^; KH_2_PO 170 mg l^−1^; KNO_3_∶ 969.5 mg l^−1^; MgSO_4_∶ 180.5 mg l^−1^ and NH_4_NO_3_∶ 1650 mg l^−1^, and micronutrients: CoCl_2_.6H_2_O : 0.025 mg l^−1^; CuSO_4_.5H_2_O : 0.025 mg l^−1^; FeNaEDTA 36.7 mg l^−1^; H_3_BO_3_∶ 6.2 mg l^−1^; KI 0.83 mg l^−1^; MnSO_4_.H_2_O : 16.9 mg l^−1^; Na_2_MoO_4_.2H_2_O : 0.25 mg l^−1^; ZnSO_4_.7H_2_O : 8.6 mg l^−1^ and KNO_3_∶ 930.47 mg l^−1^. As signal molecules are unstable at high temperature and cannot bear sterilization, axenic conditions were ensured by enclosing non sterile casts from earthworm husbandry and control soil into a 0.22 µm mesh size nylon membrane (MAGNA, Nylon, Transfer membrane, GE Water & Process Technologies, U.S) which let small molecules to diffuse into the gel while keeping bacteria inside. Seeds of *Oryza sativa* L. cv Morobekan were provided by the Laboratoire de Semences et des Ressources Biologiques of the Centre de Ressources Biologiques Tropicales de Montpellier CIRAD (France) and seeds of *Lolium perenne* L. bought in garden center. They were sterilized for 5 minutes in Teepol HB7 (Sigma Aldricht, Germany) and 10 minutes in ethanol 70°. *O. sativa* and *L. perenne* were grown respectively for 18 and 9 days in an *in vitro* culture chamber under controlled conditions at respectively 30°C and 19°C, with a 12-hours photoperiod (light intensity: 200 µmol photons s^−1^). There were two independent experiments and ten biological replicates per treatment (either with control soil or earthworm casts).

### Microcosm Experiment with Mutants

All *Arabidopsis thaliana* seeds were provided by the Arabidopsis Biological Resource Center (ABRC) at The Ohio State University (U.S.A.). As auxin-like compounds were the best candidates for the signal molecules involved in the earthworm effect [19], [20], [49], we set up a microcosm experiment, to compare the response of *A. thaliana* cv Colombia wild type and double mutant *aux1-7;axr4-2* (NASC ID: N8040, http://www.arabidopsis.org) in the presence or absence of earthworms. The *AUX1* gene is coding for an auxin influx facilitator protein [33]. The *AXR4* gene is involved in the polar localisation of the protein encoded by *AUX1*, and is thus responsible for the polarised auxin transport from shoot apex to root tips [34], [35]. As a consequence, auxin concentration in the root cells of the mutant is lower than in the wild type, which is responsible for an altered root growth and a decreased primary production, observed with the dwarf phenotype [32]. If earthworms are producing auxin-like compounds in the soil, these molecules could enter root cells, increase intracellular auxin concentration, and restore the altered phenotype of the double mutant.

*A. thaliana* cv Columbia wild type and the double mutant *aux1-7;axr4-2* were grown in experimental units of PVC (10 cm diameter, 15 cm height, n = 5 per treatment) filled with 1 kg of dry soil, which was then maintained at 80% of the field capacity. One week after soil humectation, five earthworms (1.88 g ±0.09) were introduced. Two weeks after earthworm introduction, one seed of wild type or mutant was sown in each experimental unit. Plant growth was carried on for 23 days in growth chamber under controlled conditions (Conviron, Canada): 20±1°C and 15±1°C day and night temperatures, 55% ±5% relative humidity, 200 µmol m^−2^ s^−1^ PPFD for 10 h per day.

### Transcriptome Analysis

#### Experimental protocol

Two experiments were made at one week of delay to ensure robust and repeatable results. Plants were grown in experimental PVC units (20 cm diameter, 16 cm height), filled with 6 kg DW soil and maintained at 75% of the field capacity. Seven earthworms (for a total of 3 g on average) were introduced. After one week, five seeds of *A. thaliana* cv Columbia were sown per microcosm and grown in growth chamber under controlled conditions (Conviron, Canada): 20±1°C and 18±1°C day and night temperatures, 70% ±5% relative humidity, 200 µmol m^−2^ s^−1^ PPFD for 10 h per day. Plants were harvested 42 days after sowing, when their rosette is 80% of their final size, corresponding to the 3.80 growth-stage as defined by [50].

### RNA Extraction and Microarray Analysis

Plants were pooled according to the treatment [51]. RNA was extracted from rosettes and roots using RNeasy Plant Mini Kit (Qiagen, France) with an on-column DNase digestion using DNase I (Qiagen, France). The quality of the RNAs was assessed with the Agilent Bioanalyser (Agilent, Santa Clara, U.S.A.) and the quantity determined with Ribogreen (Invitrogen, Carlslab, U.S.A.). cRNAs were produced with the “Message Amp aRNA” kit (Ambion, Austin, U.S.A). Five µg of cRNAs were reverse transcribed in the presence of 200 U Superscript II (Invitrogen, Carlslab, U.S.A.), cy3-dCTP and cy5-dCTP (NEN, Boston, U.S.A.) and hybridized on Complete Arabidopsis Transcriptome MicroArrays (CATMA), each with 24576 Gene Specific Tags from *A. thaliana* [52], with a dye swap to avoid dye bias [13]. After an array-by-array normalization, a global intensity-dependent normalization and a correction of a print-tip effect on each metablock, paired t-tests were performed on the logarithm base 2 of the ratio of transcript abundance. The raw P-values were adjusted by the Bonferroni method. Transcript abundance of 104 genes was increased in the first experiment and 103 in the second one, with 59 genes in common (Table 2). We looked for these 59 genes in Genevestigator database and we found 56 of them for which we have observed an increase in transcript abundance in the presence of earthworms (in column). As it is not possible to export rough data from Genevestigator, we selected the 60 studies among the 54 922 referenced (in line) exhibiting the most similar directional changes with the 56 genes of our study by choosing the “mosaic” with the highest number of red squares (higher transcript abundance in the treatment as compared with control).

### Real-time PCR Validation

First strand cDNA from leaves and roots of *A. thaliana* were synthesized by the reverse transcription of 2 µg of total RNA using an oligo-dT_(15)_ primer, Protector RNase Inhibitor (Roche, France) and Omniscript Reverse Transcriptase kit (Qiagen, France). Real-time PCR was performed in a LightCycler 2.0 system (Roche Diagnostics, France) with the qPCR mastermix LightCycler® FastStart DNA MasterPLUS SYBR Green I (Roche Diagnostics, France). Primers of three constitutive genes (At5g11770, At3g18780 and At5g46290) as well as underexpressed and overexpressed genes (At5g59310, At5g47220, At1g28370, At3g04720, At3g23550, and At1g73260) were designed. The amplification program was made of an initial denaturation at 95°C for 10 min, 35 cycles of amplification at 95°C for 20 s, followed by 56°C for 20 s and 72°C for 20 s. The transcript abundance was standardized with At5g11770 taken as reference with the geNorm v3.5 software. A logarithm base 2 normalization of transcript abundance was performed and results (Figure 3) show similar direction in the relative transcript abundance for real-time PCR and micro-arrays.

### Root System Analysis

Root length, average diameter, root surface, number of forks and tips were analyzed with a digital scanner (EPSON Expression 10000 XL, Epson America Inc., U.S.A.) coupled with the WinRHIZO software (WinRHIZO, version 2007 pro, Regent Instrument, Canada), following recommendations found in literature [53]: a resolution of 16 p mm^–1^ (400 dpi), with the automatic transformation threshold and a double light system.
